# A Self-Switchable Polymer Reactor for Controlled Catalytic Chemistry Processes with a Hyperbranched Structure

**DOI:** 10.3390/ma11020245

**Published:** 2018-02-06

**Authors:** Rong Luo, Hong Yang, Xiaobo Deng, Liqiang Jin, Yulu Wang, Songjun Li

**Affiliations:** 1 School of Leather Chemistry and Engineering, Qilu University of Technology (Shandong Academy of Sciences), Jinan 250353, China; yh1452147@163.com (H.Y.); jin-liqiang@163.com (L.J.); wangyl810706@126.com (Y.W.); 2 Shandong Key Laboratory for Testing Technology of Material Chemical Safety, Jinan 250102, China; sdzjy0339@126.com; 3 School of Materials Science & Engineering, Jiangsu University, Zhenjiang 212013, China

**Keywords:** polymer reactor, hyperbranched, metal nanoparticles, switchable catalysis

## Abstract

A self-switchable polymer reactor with a hyperbranched structure for controlled catalytic chemistry processes is reported. This polymer reactor was made of silver nanoparticles and a polymer carrier consisting of hyperbranched polyethylenimine and hydroxyethyl acrylate that behaved as thermally switchable domains. Below the transfer temperature, relatively strong catalytic reactivity was demonstrated due to the leading role of hydrophilic groups in the switchable domains, which opened access to the substrate for the packaged silver nanoparticles. In contrast, it showed weak catalysis at relatively high temperatures, reducing from the significantly increased hydrophobicity in the switchable domains. In this way, the polymer reactor displays controllable, tunable, catalytic activity based on this approach. This novel design opens up the opportunity to develop intelligent polymer reactors for controlled catalytic processes.

## 1. Introduction

There are fascinating prospects for metal nanoparticles (NPs) due to their significance in widespread applications and particularly in catalytic applications. Rapid evolution in nanotechnology and intelligent materials offer opportunities to exploit functional metal nanoparticles. A clever design combining catalytic metal nanoparticles with advanced materials can render metal nanoparticles with new functions, typically like controllable catalytic performance. This field is further consolidated through smart polymers as carriers to stimulate responses [1,2]. Among them, polymer reactors represented by poly(N-isopropylacrylamide) (PNIPAm) substrate/metal nanoparticle active ingredient are the earliest and most successful examples [3]. The principle is that PNIPAm has a special phase change low critical solution temperature (LCST), resulting in the swelling or shrinking of the polymer network, such a structural transition results in either the wrapping of metal nanoparticles inside the polymer substrate with quenched or reduced catalytic activities, or releasing the metal nanoparticles from the polymer substrate with much enhanced catalytic activities. Based on this mechanism, the PNIPAm-based polymer reactors realize the controllable and tunable catalytic functionality.

However, in most cases such polymer reactors with an inversable response do not have high potential for practical applications in controllable catalysis [4,5,6], simply because PNIPAm has a low phase transition temperature and a moderately adjustable temperature range that does not meet the wide range of chemical reactions. In addition, all the current polymer reactor substrates have linear structures, and cannot meet the needs of many complicated reactions, thus bringing in apparent barriers for the develop of polymer reactors.

Unlike the linear intelligent polymer materials, hyperbranched polymer (HBP) substrate possesses advantages in performance such as low viscosity, good solubility, good weatherability, high reactivity, etc. [7,8,9]. Therefore, it is a rare substrate material for polymer reactors. Unfortunately, stimuli-responsive polymer reactors with such a hyperbranched structure still remain underdeveloped.

There are three strategies for preparing thermosensitive hyperbranched polymers. The first is incorporating temperature-sensitive groups or polymers onto the surface of hyperbranched polymers [10]. Second, by introducing hydrophilic or hydrophobic functional groups onto the molecular surface or inside, the temperature sensitivity is imparted to the hyperbranched polymer by the relative balance of the hydrophilic and hydrophobic moieties [11,12]. Finally, by constructing the backbone of the hyperbranched polymer with temperature sensitivity [13].

**Scheme 1:**
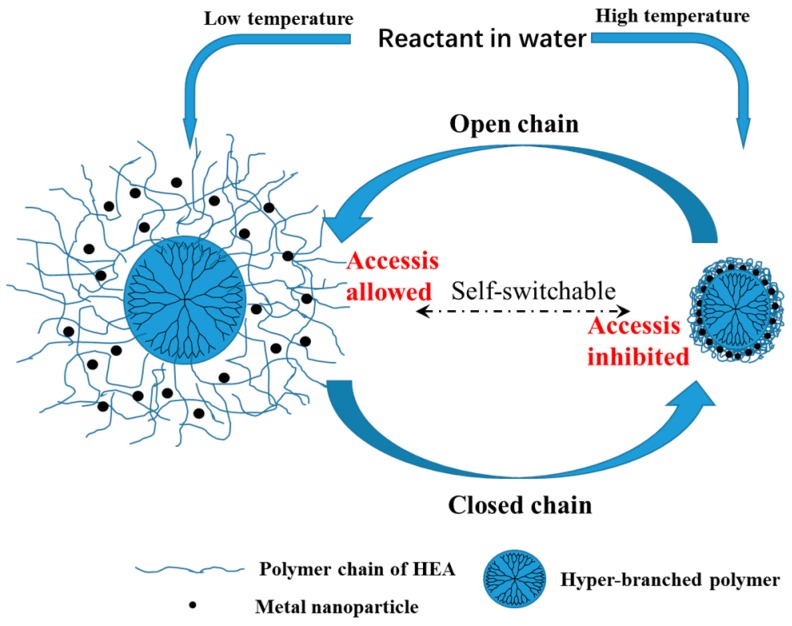
Proposed mechanism for the AgHBP-A reactor.

Inspired by this elegant work, we herein report a first temperature-sensitive HBP reactor (namely AgHBP-A) with a controllable catalytic characteristics, better fluidity and solubility in aqueous solution. This study uses a common and convenient monomer to synthesize the HBP reactor. Firstly, ethylenediamine and methyl acrylate are employed as the basic material to synthesize hyperbranched polyethylenimine using the Ax + By monomer synthesis method, and then its surface is modified with hydroxyethyl acrylate to obtain a smart HBP polymer substrate with rich hydrophilic hydroxyl groups and hydrophobic saturated aliphatic hydrocarbons, and finally a HBP reactor AgHBP-A with tunable properties is obtained with Ag NPs as the active ingredient. This study shows that at temperatures lower than the critical temperature (e.g., 30 °C), the prepared HBP reactor AgHBP-A has a higher catalytic efficiency because of a large number of hydroxyl groups on the surface of the polymer microspheres; other hydrophilic groups formed hydrogen bonds, resulting in the swelling of the polymer network, which allows reactants to access the encapsulated Ag NPs. After their phase transitions to hydrophobicity upon changes of increased external temperature (e.g., 55 °C), broken hydrogen bonds cause the polymeric building to be insoluble in water, and the polymer network shrinks, which largely restricts the access of reactants to the encapsulated Ag NPs, thereby causing dramatically decreased catalytic activity. Such hydrophilic groups and hydrophobic saturated aliphatic hydrocarbons within polymeric networks would allow better control of reactants’ access to the Ag NPs, thereby benefiting the access-regulated reactivity, as proposed in Scheme 1.

For a comparative study, two control samples, namely HBP-A and AgHBP-N, were also prepared under comparable conditions. AgHBP-N is a nonresponsive polymer reactor with the substrate made of hydroxyethyl acrylate. HBP-A has the same polymer carriers structure as AgHBP-A, but no Ag NPs are included in its HBP architectures (herein, AgHBP represent the hyperbranched polymer reactor including Ag, the ‘N’ suffix means the ‘non-responsive’ properties in contrast to the ‘A’ suffix means ‘activity’, switchable characteristics in other catalysts). For a convenient discussion, all of the prepared polymer reactors and carriers were mentioned afterward as the conceptual catalysts. In order to investigate the reactivity of these reactors, a classic model reaction of reducing methylene blue (MB) with NaBH_4_ was selected, as previously described [14,15]. The objective of this study is to demonstrate that smart reactors designed with HBPs possess controllable and self-switchable catalytic properties.

## 2. Experiment Section

*Synthesis of AgHBP-A*. Pre-AgHBP-A (14.0 g) (see Appendix A) and silver nitrate (5.35 g) were dissolved in 50 mL water. After being dispersed with sonication and nitrogen for 4 h, the encapsulated ionic silver was then reduced by an excess of sodium borohydride (tenfold, with regard to ionic nickel). The resulting polymer reactor was thoroughly washed with ethanol and water, and then dried under flowing nitrogen [16]. In this way, this novel HBP catalyst (i.e., AgHBP-A) was prepared.

*Synthesis of HBP-A and AgHBP-N*. Two controls samples, as previously mentioned, named “HBP-A”, “AgHBP-N”, were also prepared under comparable conditions. During the preparation of AgHBP-N, hyperbranched polyethylenimine was replaced with the same amount (in mole) of hydroxyethyl acrylate (HEA). HBP-A was the polymeric carrier of AgHBP-A and prepared without using Ag.

### 2.1. Characterization

The Fourier Transform Infrared Spectroscopy (FT-IR) were obtained using a FT-IR apparatus (Nicol-let MX-1E, Wisconsin, USA). The Nuclear magnetic resonance hydrogen spectrum (^1^H NMR) were recorded by nuclear magnetic resonance (Bruker, 300 MHz, Karlsruhe, GER).

Approximately 2 µL of the diluted particle suspension was dried on a carbon-coated copper grid and the Transmission electron microscopy (TEM) images of the prepared catalysts were observed by field emission transmission electron microscopy (JEOL Ltd., JEM-2100, Tokyo, Japan) operated at 200 kV.

X-ray diffraction patterns (XRD) (Rigaku, Tokyo, Japan) of the samples recorded at room temperature, which equipped with Ni-filtrated Cu Kα radiation (40 kV, 200 mA, λ = 1.5406 A) at 10–80° with a scanning step of 0.02°/0.2 s.

### 2.2. Self-Switchable Interactions

The self-switchable interactions of HBP reactor were studied as a function of temperature, by using dynamic light scattering (DLS) (Bettersize 2000, Dandong, China). For equilibrium, all the samples concerned were kept at the specified temperatures for at least 10 min before acquiring hydrodynamic radius (*R_h_*). The diameters of dried samples are measured in ethanol, and the reversible volume phase transition can be expressed with swelling rate (*R_c_*). The phase transition mechanism of the polymer reactor in aqueous solution is obtained by comparing responsive catalyst and nonresponsive AgPC-N [3]:

(1) $$R_{c}=\left[{\left(\frac{R_{h}-R_{d}}{R_{d}}\right)}_{S}-{\left(\frac{R_{h}-R_{d}}{R_{d}}\right)}_{N}\right]\times 100\%$$

Here in, *R_h_* is the hydrodynamic radius of particles, *R_d_* is the particle size of dried particles, ‘*S*’ means the self-switchable catalysts and ‘*N*’ indicates the non-responsive catalyst.

### 2.3. Catalysis Test

The catalytic properties of the polymer catalysts were evaluated using the reduction of methylene blue in the presence of sodium borohydride [14,15]. The substrate (MB) was added into 2 mL NaBH_4_ aqueous solution with the initial concentration 2.6 μmol·mL^−1^ (total volume: 4 mL) (NaBH_4_, 20 folds with regard to the substrate). The solid content of the polymer catalysts used in every test was 1.5 mg·mL^−1^. The reduction of the substrate was monitored spectrophotometrically. The reactivity of these polymer catalysts was obtained from the average of three runs.

### 2.4. Electrochemical Tests

Electrochemical tests were further performed to interrogate the catalytic mechanism between the prepared polymer catalysts and substrate [17]. Three-electrode cyclic voltammetry (CV) is performed with an Au-plate working electrode, Pt-wire counter electrode and Ag/AgCl ref. electrode (CHI760E, Shanghai, China), polymer catalysts (10.0 mg) that pre-absorbed ca. 50 nmol substrate (i.e., MB) were placed into a cuvette encircled by a diffusion-eliminating sonication apparatus (supporting electrolyte: 0.01 mmol·mL^−1^ KCl; 10 mL). The desorption behavior of the substrate is continuously scanned with CV until a stable desorption/reduction profile is obtained (scanning range, 0.2~−0.9 V; rate, 1 mV·s^−1^).

## 3. Results and Discussion

### 3.1. ^1^H NMR and FT-IR Analysis

**Figure 1:**
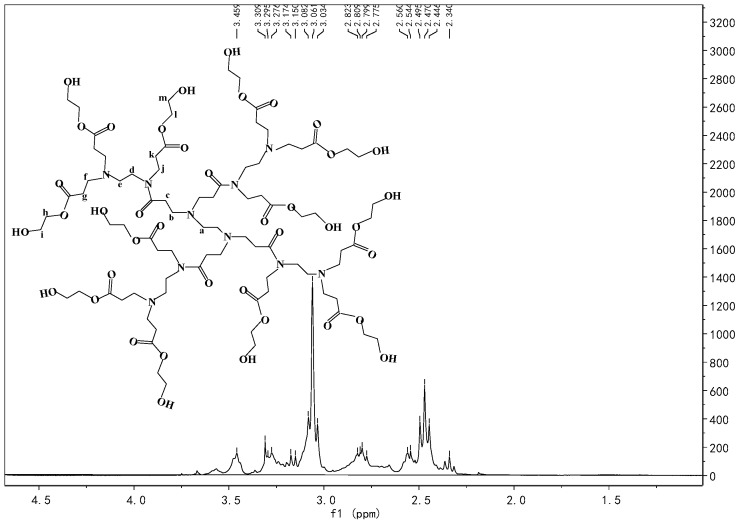
^1^H NMR spectra of the hyperbranched polymer reactor.

^1^H NMR and FT-IR analyses were first used to characterize the composition and structure of the HBP reactor. ^1^H-nuclear magnetic resonance spectroscopy (300 MHz, D_2_O, 298 K) (Figure 1). δ: 2.56 (m, br, OHCH_2_CH_2_OOCCH_2_C*H*_2_)_2_N–), 2.81~2.82 (m, br, (OHCH_2_CH_2_OOCC*H*_2_CH_2_)_2_NCH_2_CH_2_NCH_2_C*H*_2_COOCH_2_–), 3.459 (m, br, (OHCH_2_C*H*_2_OOCCH_2_CH_2_)_2_NCH_2_CH_2_NCH_2_CH_2_–COOC*H*_2_CH_2_OH), 3.276~3.309 (m, br, (OHCH_2_CH_2_OOCCH_2_–CH_2_)_2_NC*H*_2_CH_2_NCH_2_CH_2_COOCH_2_–), 3.034~3.028 (m, br, (OHC*H*_2_CH_2_OOCCH_2_CH_2_)_2_NCH_2_CH_2_NCH_2_CH_2_COOCH_2_C*H*_2_OH). There is no chemical shift of the hydroxyl group H in the structural formula. These chemical shifts were complex due to the complicated composition, many peaks overlap, so it is not easy to distinguish. Nevertheless, the structure of the product was judged by comparison with intermediate-2 of HBP. Thus, this HBP reactor was prepared in the desired form. Details of other architecture elucidation determinations can be found in the Appendix A.

**Figure 2:**
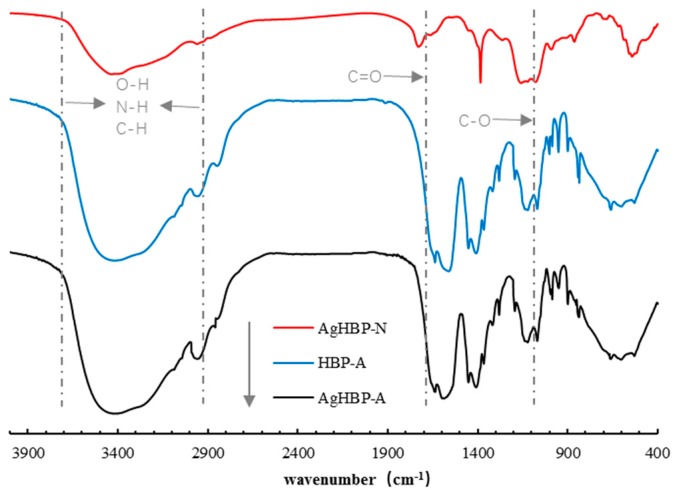
FT-IR spectra of the prepared polymer reactors.

Figure 2 presents the FT-IR spectra of the prepared polymer reactors. They included the two control sample spectra. HBP-A exhibited almost the same spectrum as AgHBP-A, whereas that of AgHBP-N is different from the above. The same spectra between HBP-A and AgHBP-A can be ascribed to the comparable composition between HBP-A and the polymer carrier of AgHBP-A. The difference between AgHBP-A and AgHBP-N at 1000–1500 cm^−1^ may be ascribed to the presence of the hyperbranched polyethylenimine within AgHBP-N. In the spectrum of AgHBP-A, at 3000~3700 cm^−1^ belongs to the stretching vibration absorption of O–H/N–H, at ~1660 cm^−1^ was the stretching vibration absorption of C=O, at 1460 cm^−1^ and 1410 cm^−1^ was the in-plane bending and deformation vibration absorption of C–H, respectively. At 1150 cm^−1^ it belongs to the stretching vibration absorption of C–O in COO–, and ~1080 cm^−1^ belongs to the stretching vibration absorption of C–O in –CH_2_–OH. It is therefore clear that the prepared AgHBP-A was expected.

### 3.2. TEM and XRD Analysis

**Figure 3:**
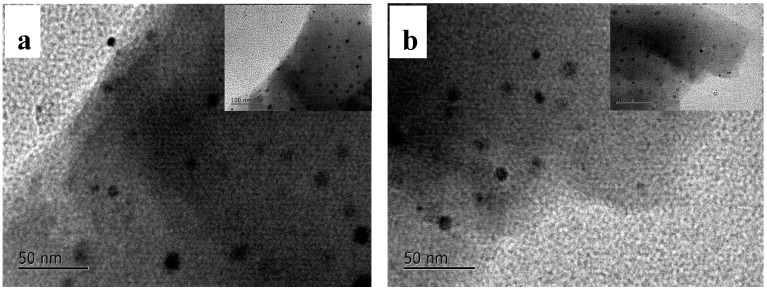

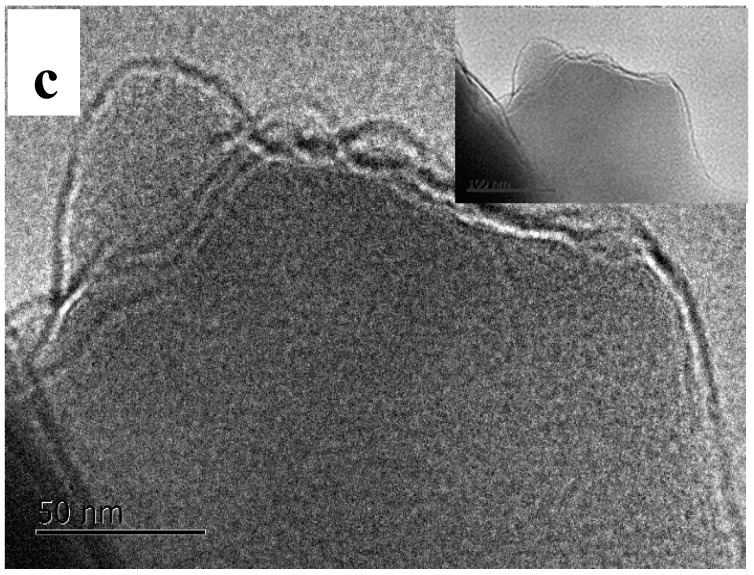
TEM images of the prepared polymer reactors. (**a**) AgHBP-A; (**b**) AgHBP-N; (**c**) HBP-A.

**Figure 4:**
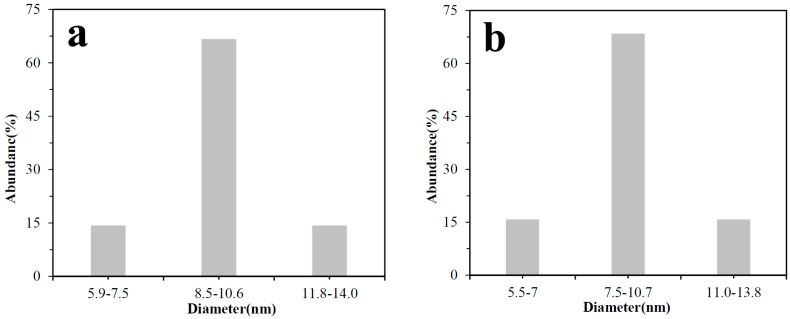
Particle distribution of AgHBP-A (**a**) and AgHBP-N (**b**).

The polymer reactor AgHBP-A was constructed from Ag NPs and HBP carrier. Figure 3 presents the TEM images exhibiting the morphology of metal nanoparticles encapsulated in the prepared polymer reactors. The mean size of Ag NPs is 9.84 (AgHBP-A) nm and 9.41 nm (AgHBP-N) respectively estimated from the TEM images. Figure 4 further demonstrates the particle distribution of the as-prepared reactor. All results confirm that these polymer catalysts have been successfully prepared in the desired form. Besides, it is an easy method to create a nano-architecture composed of hyperbranched polymer materials.

**Figure 5:**
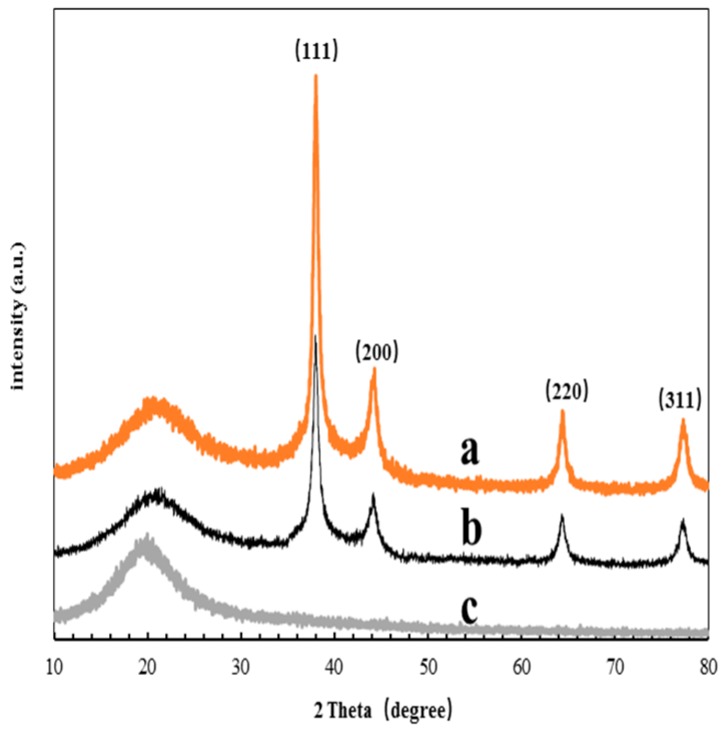
XRD patterns of the prepared polymer reactors. (**a**) AgHBP-A; (**b**) HBP-A; (**c**) AgHBP-N.

An XRD pattern was used to investigate the Ag NPs contained in the prepared polymer catalysts. The characteristic diffraction peaks of Ag from the {1 1 1}, {2 0 0}, {2 2 0}, and {3 1 1} planes were found obviously, which indicates the formation of the face-centered cubic silver (Figure 5). Just as expected, the XRD patterns of HBP-A showed peaks at the same 2theta values with AgHBP-A. However, no peaks were observed in AgHBP-N (except for polymer peaks), which agreed with the absence of silver metal.

### 3.3. Evaluation of the Self-Switchable Interaction

**Figure 6:**
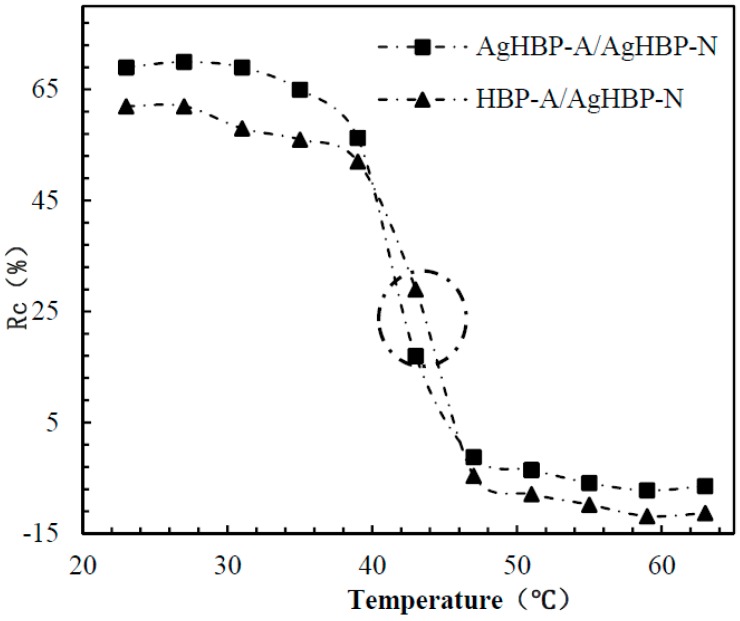
DLS curves of the prepared polymer reactors. (The circle means the LCST of AgHBP-A).

The DLS curves of these prepared polymer catalysts are shown in Figure 6. Compared with AgHBP-N (i.e., the non-responsive catalyst), *R_c_* values of the AgHBP-A and HBP-A revealed a significant dependence on temperature, especially in the range of 38–48 °C which is the LCST value of HBP 43 °C (Mark with a small circle). The *R_c_* of AgHBP-A and HBP-A decreased with elevated temperature and the major decrease appeared at 38–48 °C. Below this region, AgHBP-A and HBP-A revealed a high *R_c_* associated with the formation of hydrogen bonds in the aqueous phase of the hydrophilic groups, which opened the swelling of the polymeric building blocks (Scheme 1). In contrast, when the temperature elevated from 38 °C to 48 °C, the *R_c_* of AgHBP-A and HBP-A dramatically decreased in response to the cleavage of hydrogen bonds resulting in the dominant role of hydrophobic groups, which caused shrinking of the polymeric microsphere. This result indicates the self-switchable properties of AgHBP-A and HBP-A, as expected.

### 3.4. Switchable Catalysis

**Figure 7:**
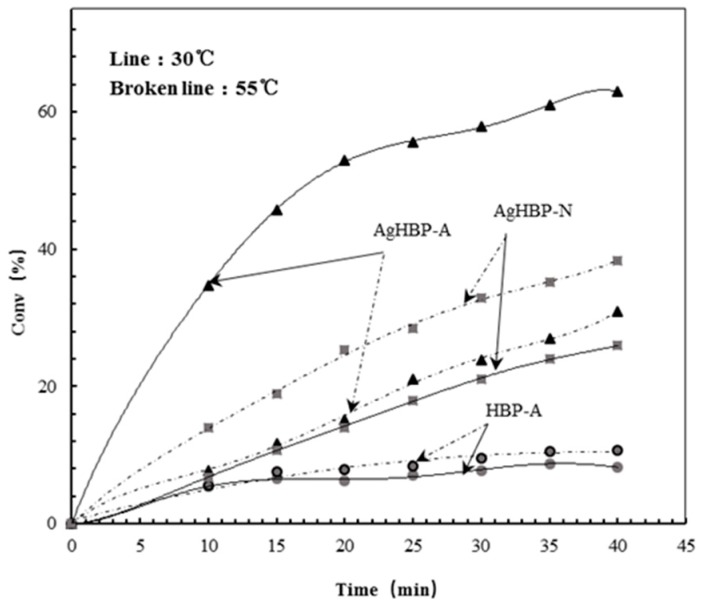
Reactivity of the prepared polymer reactors for the reduction of MB.

The catalytic properties of the prepared HBP reactors was evaluated using the reducing reaction of MB with NaBH_4_ catalyzed by Ag NPs. Two representative temperatures, 30 °C and 55 °C, either higher or lower than the transition temperatures of AgHBP-A (43 °C), were selected to scrutinize the self-switchable catalytic behaviors. As shown in Figure 7, HBP-A did not exhibit essential catalytic ability due to the lack of catalytic Ag NPs. The non-responsive AgHBP-N showed lower reactivity at 30 °C, and the catalytic ability was slightly enhanced at 55 °C. The reason for this was that AgHBP-N has a certain polymer swelling property in the aqueous solution, and the access between the metal nanoparticles and substrate is slowly “opened” with elevated temperature and the catalytic activity is gradually increased. In contrast, at 50 °C, AgHBP-A revealed a weak catalytic ability, much lower than AgHBP-N. We believe that the hydrogen bond rupture caused the polymer network to shrink. Ag NPs were encapsulated in the polymer building, the access between the metal nanoparticles and substrate was “closed” and the catalytic activity was hindered. However, at 30 °C, AgHBP-A showed good catalytic efficiency and demonstrated a significant reactivity, which was formed by the hydrophilic action that caused the polymer network to swell, Ag NPs to be released from the polymer capsule, the access to “open”, and the catalytic activity to open. This result, as previously explained, can be associated with the switchable interaction within the AgHBP-A. Hydrophilic and hydrophobic interactions among the HBP architecture controlled access to the catalytic sites of Ag NPs, thereby leading to the generation of the self-switchable catalytic ability.

### 3.5. Kinetic Study of Catalysis

A pseudo-first-order kinetic study on the concentration of MB evaluated the catalysis; since the concentration of NaBH_4_ significantly overexposed that of MB, the reaction rate could be assumed to be independent of the borohydride concentration. The classical derivation process is reflected in many previous works [18,19], the formula of conclusion is:

(2) $$\ln\left(1-x_{H}\right)=R_{r}\;\ln\left(1-x_{L}\right)$$

**Figure 8:**
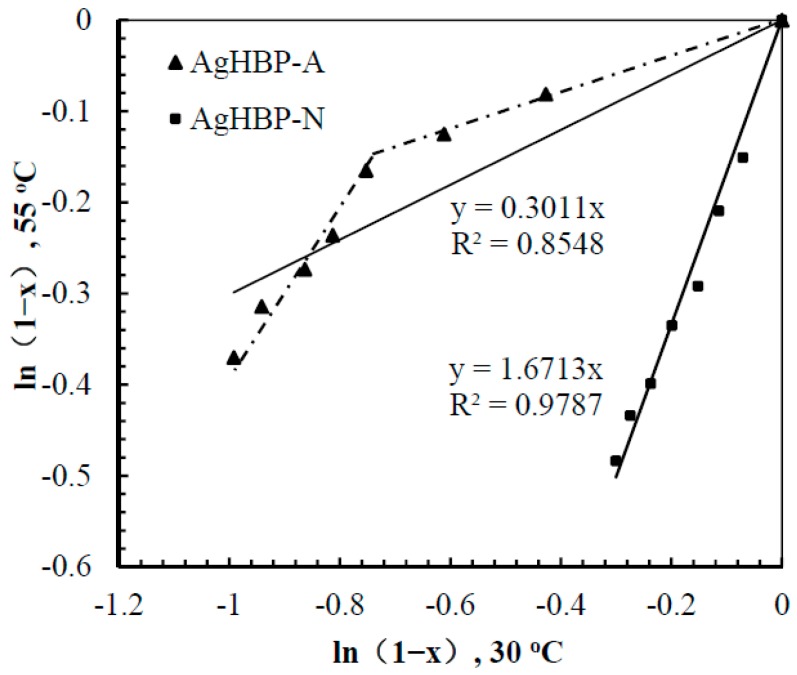
Kinetic fitting of the catalytic activities of the prepared polymer reactors (MB initial concentration, 2.6 μmoL·mL^−1^; polymer reactors, 1.5 mg·mL^−1^).

Here, *x* is the conversion of MB at time t; the subscripts ‘*L*’ and ‘*H*’ represent the relatively low and high temperatures, respectively; and *R_r_* (≡*k_H_*/*k_L_*) is a constant that represents the relevant reactivity, which can be achieved by plotting the function of conversion. The catalytic relative activity of polymer catalysts at different temperatures is associated with the conversion. As shown in Figure 8, the linear correlative coefficient of the Rr values in the AgHBP-N system was as high as 0.9787, exhibiting good linear correlativity. This is due to the rise in temperature caused when the AgHBP-N system reactivity increases (67% increase). In contrast, the catalytic activity of AgHBP-A was significantly different from that of AgHBP-N. With the increase of temperature, the catalytic activity revealed a negative growth (and ran with bending). The catalytic activity of AgHBP-A was lower than that of 30 °C at 50 °C, that is, the catalytic activity decreased with the increase of temperature. This result strongly indicates that catalysis by the AgHBP-A is a tunable process, which is tunable with temperature. Kinetic fitting of the catalytic activities of the as-prepared reactor is further reflected in the temperature response and the self-switchable properties within AgHBP-A.

### 3.6. Dynamic Binding Behavior

**Scheme 2:**
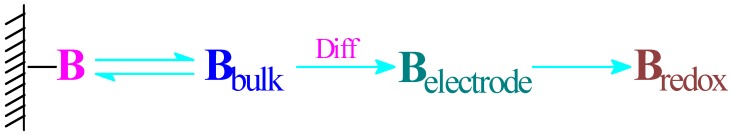
Schematic presentation of an electrochemical process with binding molecule B.

**Figure 9:**
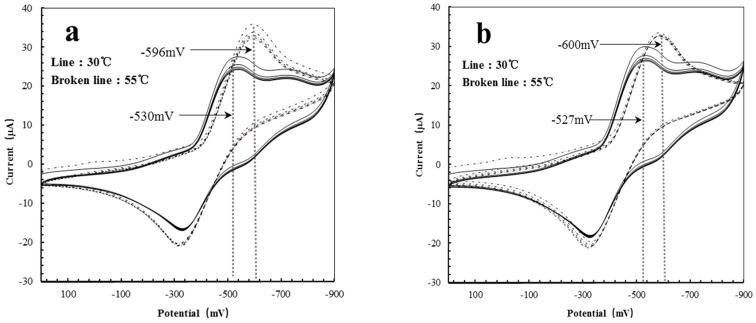

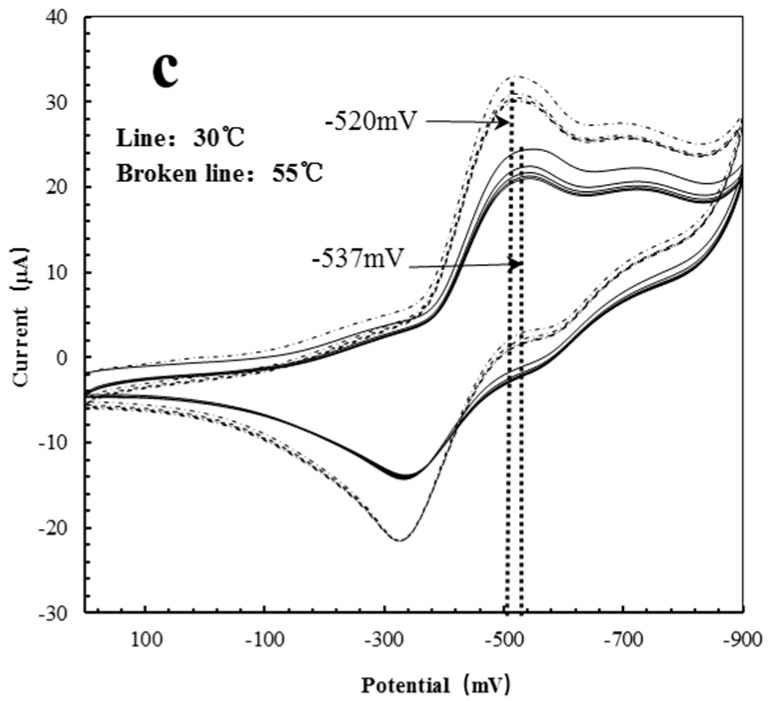
Reduction profiles with MB desorbing from the prepared polymer reactors ((**a**) AgHBP-A; (**b**) HBP-A; (**c**) AgHBP-N).

Electrochemical studies were further performed to ascertain the switchable catalytic behaviors of the prepared polymer catalysts. The underlying issue for polymer catalysts and their catalytic mechanisms lies in the interaction between the catalyst and the substrate. It is known that the oxidation or reduction potential of the bonded molecules depends on the bonding state. Stronger binding will need more energy to overcome the binding. The theory and details, as outlined in Scheme 2, have been widely described [20,21]. In detail, the substrate (B) in the electrochemical system involve the desorption, the diffusion to the electrode surface and electrochemical terminal reaction. In the event that the diffusion is eliminated with sonication, the binding constant (K) can be directly related to the change of the redox potential (E) (i.e., Δln K = αΔE). As such, the electrochemical studies were performed in accordance with the paradigm, as shown in Figure 9.

Given the responsiveness of AgHBP-A, 30 and 55 °C were again selected for a comparison. MB attached to AgHBP-A at 30 °C exhibited a desorption/reduction peak at −530 mV. In contrast, this peak shifted to a larger position (−596 mV) at 55 °C (cf. Figure 9a). As anticipated, the AgHBP-A demonstrated a stronger interaction with MB at 55 °C than at 30 °C. The self-switchable ability appears to play some role for the hydrophilic-hydrophobic interaction.

To further address the interaction, we provide the desorption/reduction potentials of all the prepared HBP reactors with the reactant MB (cf. Table 1). Despite the encapsulated Ag NPs, AgHBP-A showed a desorption/reduction potential almost comparable to HBP-A. Both of them revealed comparable potential changes (−66 mV and −73 mV) in response to the elevated temperature. In contrast, the desorption/reduction peak of the conventional AgHBP-N shifted to the opposite direction with enhanced temperature and did not raise significant potential changes (+17 mV). This slight change indicated that the interaction between AgHBP-N and the reactant MB is approximate at two selected temperatures and does not have thermal response characteristics. Clearly, this change is caused by the swelling of the polymer. Essentially, the self-switching catalysis of AgHBP-A is the result of the heat-responsive HBP support. That is, the HBP carrier is the driving force for AgHBP-A to have catalytic controllability. The principle is to control the access to the catalytic sites of silvers through a responsive HBP carrier for the purpose of switchable catalysis.

## 4. Conclusions

An “active” and self-switchable polymer reactor with a hyperbranched structure is reported in this study. This HBP reactor was constructed from Ag NPs and a polymer carrier consisting of hyperbranched polyethylenimine and hydroxyethyl acrylate. The hydrophilic and hydrophobic group of molecular chains in the switchable domains acted as a molecular switch for the tuning of the access of the substrate to the encapsulated metal nanoparticles. Below the transition temperature (~43 °C), this polymer reactor revealed significant catalytic reactivity due to the leading role of hydrophilic groups in the switchable domains, which opened the access between substrate and the catalytic metal nanoparticles. In contrast, above the transition temperature, this HBP reactor revealed weak reactivity suddenly, due to the significantly increased hydrophobicity, in response to the closing of the access. In this way, this polymer reactor demonstrated the autonomic switchable catalytic ability, which provides a method for developing smart polymer reactors for controlled catalytic processes.

## Figures and Tables

**Table 1:** Reduction potentials with substrate desorbing from all the prepared polymer reactors.

Polymer Reactors	30 °C (mV)	50 °C (mV)	Delta (mV)
AgHBP-A	−530	−596	−66
HBP-A	−527	−600	−73
AgHBP-N	−537	−520	+17

**Figure A1:**
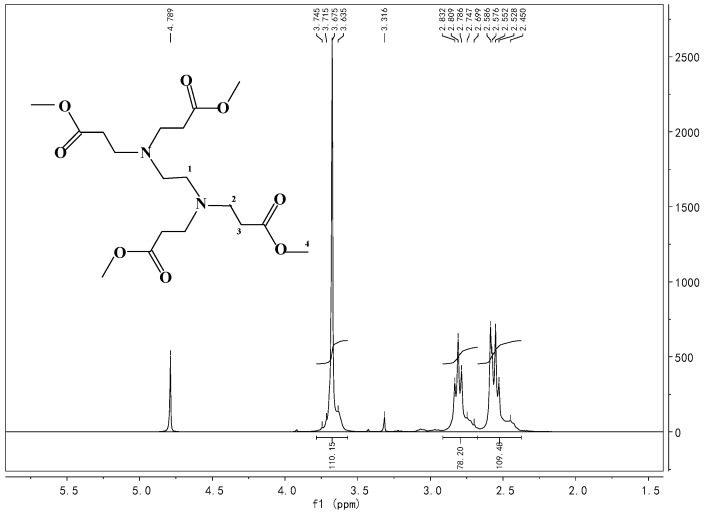
^1^H NMR spectrum of precursor of intermediate-1 of HBP.

**Figure A2:**
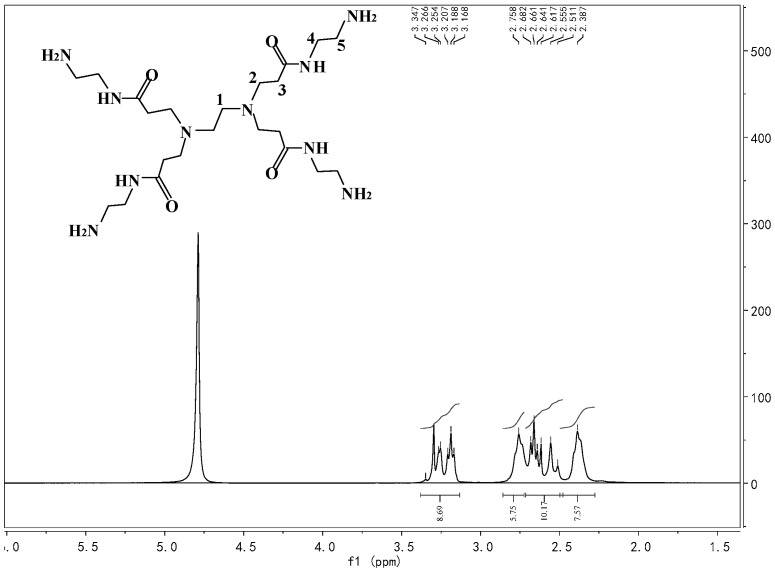
^1^H NMR spectrum of intermediate-2 of HBP.

**Figure A3:**
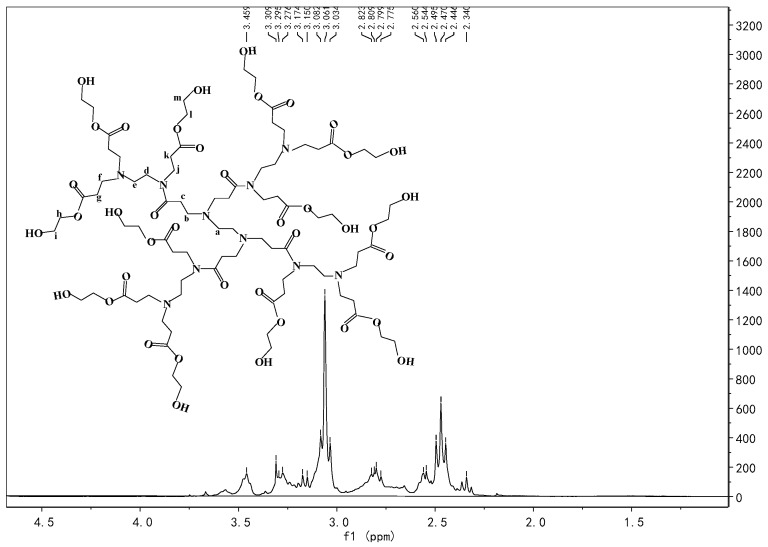
^1^H NMR spectrum of pre-AgHBP-A (AgHBP-A).

## Appendix A. Experimental Details

### Appendix A.1. Preparation of Polymer Catalysts

Unless otherwise noted, the chemicals used were of analytic grade and used as received from Sigma-Aldrich without further purification. In this study, 4A type molecular sieve activated at 300~450 °C for 4 h as a desiccant, and sealed with a dryer. Ethylenediamine (EDA) was washed with 5 wt % potassium hydroxide solution 48 h, than distilled under reduced pressure at 45~47 °C in prior to use. Methyl acrylate (MA) was washed with 5 wt % sodium hydroxide solution and dried over anhydrous sodium sulfate 24 h, finally distilled at 77~79 °C under atmospheric pressure; The polymerization inhibitor in hydroxyethyl acrylate (HEA) was removed by vacuum distillation; Methanol distilled at 64~66 °C under atmospheric pressure, adding a small amount of 4A molecular sieve sealed. Water for the reaction and analysis was collected from the Direct-Q UV System (Millipore). All reactions were made in glassware that was predried overnight at 100 °C.

### Appendix A.2. Synthesis of HBP Intermediate 1

EDA (1.803 g; 0.03 mol) was dissolved in 10 mL of methanol, then added to 100 mL of a three-necked flask equipped with a magnetic stirrer, a reflux condenser, a dropping funnel for MA solution. Before the reaction started, argon was bubbled through for 10 min to remove the air from the reaction flask bottle. Under ice-cooling, MA (20.661 g; 0.24 mol) was dissolved in 15 mL of methanol, and slowly added dropwise to a three-necked flask with a dropping funnel. About 1.5 h later, the reaction was continued at 35 °C for 36–48 h. The product was subjected to distillation under reduced pressure, washed with methanol and re-distilled to remove unreacted monomers and by-products, repeated several times, and finally dried in vacuo to give the paleyellow product HBP intermediate-1.

^1^H NMR (300 MHz, D_2_O, 298 K). δ: 2.55 (t, 8H, CH_3_OOCCH_2_C*H*_2_N–), 2.59 (s, 4H, (CH_3_OOCCH_2_CH_2_)_2_NC*H*_2_C*H*_2_ N–), 2.81 (t, 8H, CH_3_OOCC*H*_2_ CH_2_ N–), 3.68 (s, 12H, C*H*_3_OOCCH_2_–).

### Appendix A.3. Synthesis of HBP Intermediate 2

HBP Intermediate 2 was prepared using a polymerization system including HBP Intermediate 1 (9.69 g; 0.024 mol) in 20 mL methanol were put into a one-neck flask equipped with a magnetic stirrer, a reflux condenser in an ice-salt bath, argon was bubbled through for 10 min to remove the air from the reaction flask bottle before the reaction started. EDA (11.5392 g; 0.192 mol) was dissolved in 10 mL of methanol, and slowly dropwise added to a three-necked flask with a dropping funnel under stirring, About 1.5 h later, the reaction was continued at 40 °C for 36–48 h with inert gas protection. Then, the flask was fixed onto a rotary evaporator to remove the methanol and by-products under the vacuum, washed with methanol and re-distilled, repeatedly. Finally, the product dried in vacuo, a slightly yellow dope was obtained.

^1^H NMR (300 MHz, D_2_O, 298 K). δ: 2.51~2.55 (s, 4H, (NH_2_CH_2_CH_2_NHCOCH_2_CH_2_)_2_NC*H*_2_C*H*_2_N–), 2.61~2.68 (s, 8H, (NH_2_CH_2_CH_2_NHCOCH_2_C*H*_2_)_2_N–), 2.38 (t, 8H, NH_2_CH_2_CH_2_NHCOC*H*_2_CH_2_)_2_N–), 3.25~3.29 (s, 8H, NH_2_CH_2_C*H*_2_NHCOCH_2_–), 3.16~3.20 (s, 12H, NH_2_C*H*_2_CH_2_NHCOCH_2_–).

### Appendix A.4. Synthesis of Pre-AgHBP-A (HBP-A)

The precursor of polymer catalyst, as the chemical structure in Figure A3. Was based on the classic design of HBP. In detail, HBP Intermediate 2 (6.71 g; 0.01 mol) was dissolved in 15 mL of Deionized water (Ultrapure water) in a three-necked flask with inert gas protection, than slowly dropwise added the HEA (14.63 g; 0.13 mol) with a dropping funnel under stirring for 1 h. The reaction was continued at 65 °C for 24–36 h, and then precipitated with acetone. After washed with methanol under reduced pressure repeatedly and further drying in vacuum at 70 °C for 48 h, pre-AgHBP-A was obtained.

^1^H NMR (300 MHz, D_2_O, 298 K). δ: 2.56 (m, br, OHCH_2_CH_2_OOCCH_2_C*H*_2_)_2_N–), 2.81~2.82(m, br, (OHCH_2_CH_2_OOCC*H*_2_CH_2_)_2_NCH_2_–CH_2_NCH_2_C*H*_2_COOCH_2_–), 3.459 (m, br, (OHCH_2_C*H*_2_OOCCH_2_CH_2_)_2_NCH_2_CH_2_NCH_2_CH_2_COOC*H*_2_CH_2_OH, 3.276~3.309 (m, br, (OHCH_2_CH_2_OOCCH_2_CH_2_)_2_NC*H*_2_CH_2_NCH_2_CH_2_COOCH_2_–, 3.034~3.028 (m, br, (OHC*H*_2_CH_2_OOCCH_2_CH_2_)_2_NCH_2_CH_2_NCH_2_CH_2_–COOCH_2_C*H*_2_OH). There is no chemical shift of the hydroxyl group H in the structural formula. These chemical shifts were complex due to the complicated composition, many peaks overlap, so it is not easy to distinguish. In addition, the structure of the product was judged by comparison with intermediate-2 of HBP. Thus, this HBP reactor was prepared in the desired form.
